# The Rare Actinobacterium *Crossiella* sp. Is a Potential Source of New Bioactive Compounds with Activity against Bacteria and Fungi

**DOI:** 10.3390/microorganisms10081575

**Published:** 2022-08-05

**Authors:** Jose Luis Gonzalez-Pimentel, Irene Dominguez-Moñino, Valme Jurado, Leonila Laiz, Ana Teresa Caldeira, Cesareo Saiz-Jimenez

**Affiliations:** 1 Laboratorio Hercules, Universidade de Evora, 7004-516 Evora, Portugal; 2 Instituto de Recursos Naturales y Agrobiologia, IRNAS-CSIC, 41012 Sevilla, Spain

**Keywords:** Altamira Cave, bioactive compounds, *Crossiella*, predicted gene clusters, Spanish show caves

## Abstract

Antimicrobial resistance has become a global problem in recent decades. A gradual reduction in drug discoveries has led to the current antimicrobial resistance crisis. Caves and other subsurface environments are underexplored thus far, and they represent indispensable ecological niches that could offer new molecules of interest to medicine and biotechnology. We explored Spanish show caves to test the bioactivity of the bacteria dwelling in the walls and ceilings, as well as airborne bacteria. We reported the isolation of two strains of the genus *Crossiella*, likely representing a new species, isolated from Altamira Cave, Spain. In vitro and in silico analyses showed the inhibition of pathogenic Gram-positive and Gram-negative bacteria, and fungi, as well as the taxonomical distance of both strains from their closest relative, *Crossiella cryophila*. The presence of an exclusive combination of gene clusters involved in the synthesis of lanthipeptides, lasso peptides, nonribosomal peptides and polyketides indicates that species of this genus could represent a source of new compounds. Overall, there is promising evidence for antimicrobial discovery in subterranean environments, which increases the possibility of identifying new bioactive molecules.

## 1. Introduction

Numerous papers have been published on the production of antimicrobial compounds (ACs) by different bacterial groups isolated from soils and aquatic environments. There is a long tradition of using soils for isolating AC-producing bacteria since the first works of Waksman and collaborators [1]. A decade later, research focused on marine organisms [2]. At present, a considerable number of ACs were identified from terrestrial and aquatic microorganisms [3,4,5].

In 2017, the World Health Organization published a list of 12 dangerous bacteria for which new antibiotics were urgently needed [6]. Of these bacteria, nine were Gram-negative, which indicates the necessity of using antibiotics against this type of bacteria. Tacconelli et al. [7] reported that in the last two decades, only two new antibiotic classes that are active against Gram-positive bacteria have been approved by the US Food and Drug Administration and European Medicines Agency. However, in the same period, no new antibiotics against Gram-negative bacteria have been approved, and quinolones, discovered in 1962, represented the last drug recognized to be active against Gram-negative bacteria. Therefore, the search of ACs active against Gram-negative bacteria should be enhanced.

Based on the needs of the World Health Organization and the critical shortage of new antibiotics in development against multidrug-resistant bacteria, in the last few years, we have observed a growing interest in ACs produced by microorganisms thriving in caves. In fact, a number of papers have been published on the topic [8,9,10,11,12]. A vast majority of these reports were merely descriptive works on the isolation of selected cave strains that were screened for their antimicrobial properties by a spot-on lawn antimicrobial assay, and clearly more research on the nature of ACs is needed. Gohain et al. [13] published a list of actinobacterial species producing more than 60 different ACs, and Zada et al. [14] reported a list of 20 ACs produced by cave bacteria, from which 12 were excreted by species of *Streptomyces*, six by species of *Paenibacillus* and one each by *Bacillus licheniformis* and *Nonomuraea specus*. This result indicates the importance of *Actinobacteria* and *Firmicutes* as AC producers. 

The subsurface is a relatively less studied environment, and the exploration of caves as a source of ACs is relatively recent. More than 20 years ago, Groth et al. [15] reported that caves seemed to be a promising niche for finding actinomycetes producing novel ACs. This assumption was confirmed a few years later with the discovery of cervimycins, a polyketide glycoside complex produced by *Streptomyces tendae*, isolated from Grotta dei Cervi in Italy [16,17]. 

In general, most caves are unexplored or rarely visited by humans from which novel or rare taxa can be isolated [3]. This also extends to widely visited show caves [15,18]. 

As a country, Spain is rich in caves. Some cavities have a long tradition as show caves and attract a high number of visitors. In Andalusia, South Spain, 114 karstic caves and chasms were reported, and visits to caves represent a valuable economic factor for less favored areas. Cave tourism started as early as the first quarter of the 19th century in Ardales Cave [18]. Altamira Cave, the most famous Spanish cave, was discovered in 1868 and contains rock art attributed to the Upper Paleolithic Age. Early excavations at the beginning of the 20th century in Altamira, as well as further consolidation works due to the collapse of rock blocks from the ceiling, the installation of artificial lighting and massive numbers of visitors, altered the pristine ecosystem and biodeteriorated the paintings. In fact, up to 175,000 visitors were registered in 1973. As a result, the cave had to be closed in 1977, reopened in 1982 and closed again in 2002 due to the contamination of the Polychrome Hall and its paintings by phototrophic microorganisms (cyanobacteria and algae) [19]. 

Altamira and other Spanish caves were revealed as interesting niches for isolating and describing novel bacterial and fungal species [20,21,22,23,24,25,26,27]. These understudied microorganisms offer a wealth of potential for the discovery of new ACs.

Microorganisms employ a variety of strategies to compete successfully against neighbors sharing their niche. The production of ACs, as secondary metabolites, is a common strategy in oligotrophic niches. The secondary metabolites are produced by biosynthetic gene clusters (BGCs). The major classes of ACs are the Non-Ribosomal Peptides (NRP) and Polyketides (PKS), which are synthesized by large multi-enzyme complexes of NRP synthetases (NRPS) and PKS synthases (PKSs). These two classes of BGCs encompass most known antibiotics and antifungals [28,29].

A way to counteract the lack of studies on novel ACs is to characterize the bacterial genome through gene prediction and functional annotations. This permits focusing on bacteria with gene clusters of interest. Here, we report data in favor of caves as environments of increasing interest in the search for bacterial ACs.

## 2. Materials and Methods

### 2.1. Sampling, Isolation, and Identification of Bacteria

**Figure 1:**
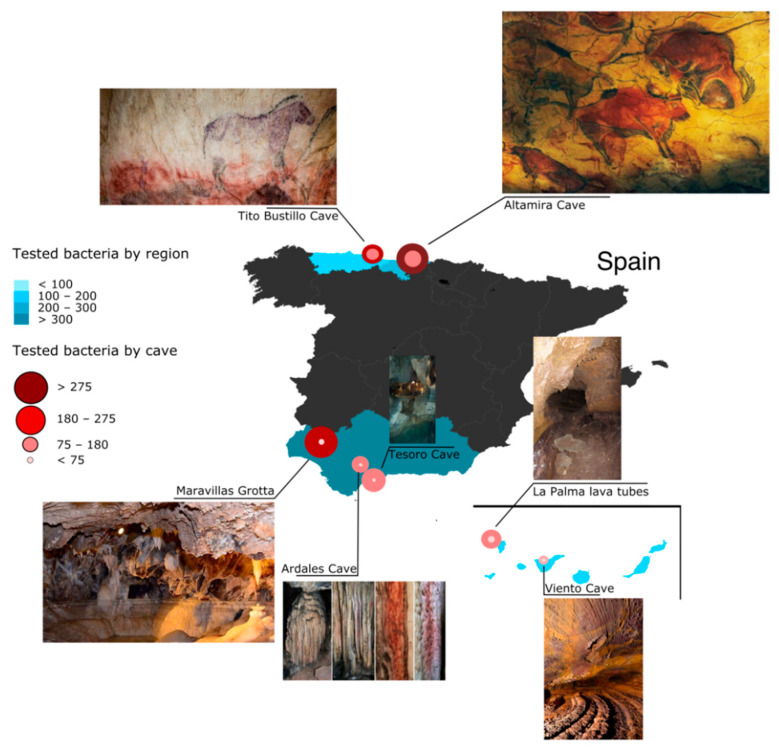
Caves sampled in Spain in search of antimicrobial compounds (ACs). Seven caves from four Spanish regions were studied. The number of strains tested by region was represented in blue. Circles represent the tested bacteria (external circles) and AC-producing bacteria (internal circles).

The samples of speleothems, microbial mats, biofilms and sediments were taken from different cave walls and ceilings from 1996 till 2020 (Figure 1). The description of the caves (geology, environmental parameters, geomicrobiology, etc.) are reported elsewhere [15,30,31,32,33]. These caves share unique environmental conditions (low and constant temperature, high relative humidity, input of organic carbon by dripping waters and visitors, etc.) which favor the development of complex microbial communities [15,19,30,31]. 

The samples were placed in sterile tubes and kept at 5 °C until arrival at the laboratory. Subsequently, they were immediately processed by suspension in saline solution and inoculation in different culture media to isolate both chemolithotrophic and heterotrophic bacteria, as reported by Groth et al. [15,16] and Jurado et al. [20,21,22,23]. The cultures were incubated at 28 °C for several weeks to allow the growth of slow-growing strains.

The air samples from the caves were collected using a high volume Surface Air System sampler (Duo SAS, model Super 360, International pBI, Milan, Italy). The samples were taken in duplicate, and the volume of filtered air was set at 100 L [32]. The culture medium was trypticase-soy-agar (TSA), and the plates were incubated at 28 °C. Morphologically different colonies were isolated in the TSA culture medium. Bacterial identification was carried out by sequencing the 16S ribosomal RNA (rRNA) gene [33]. The identification of phylogenetically related taxa was determined by the global alignment algorithm, using the EzBioCloud database [34]. The number of strains tested from each cave and the activity against pathogenic bacteria are shown in Figures S1 and S2 (Supplementary Materials).

### 2.2. Bacterial and Fungal Inhibition Assays

Of all the bacteria isolated, two strains of *Crossiella* (Cross-1 and Cross-2) were selected for further analysis. The strains were cultured in flasks with 150 mL of nutrient broth with 2% of glycerol (*v*/*v*) at 30 °C and 180 rpm. The implementation of glycerol as the carbon source promotes the synthesis of the antibiotics in actinomycetes [35,36]. After incubation, the cultures were filtered through a 0.22 µm pore size nucleopore polycarbonate filter and the filtrates were tested for antibacterial activity. The pathogenic bacteria used in this test were *Bacillus cereus* CECT 148, *Staphylococcus aureus* CECT 4630, *Escherichia coli* DSM 105182, *Pseudomonas aeruginosa* CECT 110 and *Acinetobacter baumannii* DSM 30007. In addition, an environmental bacterium, *Arthrobacter* sp., was added to the test, due to its low antimicrobial resistance. The pathogenic bacterial suspensions were prepared in 0.9% (*w*/*v*) sterile saline solution and adjusted to 0.5 McFarland units using a densitometer DEN-1B (BioSan SIA, Riga, Latvia). An inhibitory assay was carried out in duplicate in a SPECTROstar Nano microplate device (BMG Labtech, Ortenberg, Germany). The microplates were subjected to orbital shaking at 500 rpm, at 30 °C for 24 h and recorded every 30 min. 

The fungal growth inhibition assay was carried out on a solid culture of NA-Glycerol, a methodology similar to that used by Kerr [37]. The biomass of the strains Cross-1 and Cross-2 were spread over agar in a zigzag pattern and incubated at 30 °C for 24 h before inoculation of the fungal species. Once the fungi were inoculated, the plates were incubated at 25 °C for 28 days. The fungal species used in this trial were previously isolated from caves and include *Aspergillus versicolor, Penicillium chrysogenum, Cladosporium cladosporioides, Fusarium solani* and *Ochroconis lascauxensis*, some of them entomopathogens and others opportunistic pathogens [38,39].

### 2.3. Genomic Analysis

The DNA extracted for the whole genome shotgun was sequenced by Macrogen Inc. (Seoul, Korea) for both of the Illumina short-reads 150 PE, using a NovaSeq 6000 sequencer (Illumina, San Diego, CA, USA) and TruSeq DNA PCR-free for the library construction. De novo assembly was performed with SPAdes [40], using the raw reads. A pairwise genome comparison was carried out using the JSpeciesWS web tool [41]. Tetra correlation search (TCS) was deployed to look for the closest genomes, which were engaged along with both of the strains, Cross-1 and Cross-2, to calculate the Average Nucleotide Identity with BLAST (ANIb) and MUMmer (ANIm) algorithms. A phylogenomic approach was carried out through the alignment of 71 concatenated genes provided by the anvi’o platform [42]. A phylogenetic tree was reconstructed using the best substitution model predicted by MEGA XI [43]. The evolutionary history was deduced using the maximum-likelihood method and the general time-reversible model with discrete gamma distribution and invariant sites [44]. The nodes of the phylogenetic tree were well-supported with bootstrap values greater than 70. The functional characterization of the genes was obtained using Prokka version 1.14.6 [45] and antiSMASH 6.0 with strict mode and all of the extra features for the automated genome mining of secondary metabolism [46]. According to antiSMASH, the homology between sequences was fixed with a BLAST e-value below 1 × 10^−5^, with a 30% minimal sequence identity and a coverage above 25% of the sequence. The biosynthetic core genes from predicted gene clusters with antiSMASH were annotated with the UniProt database, using BLAST.

The GenBank/EMBL/DDBJ accession numbers 16S rRNA gene sequences of the strains Cross-1 and Cross-2 were ON669108 and ON669109, respectively. The sequencing data generated in this study were deposited in the National Center for Biotechnology Information (NCBI), under the BioProject ID number PRJNA769239. The whole-genome shotgun project was deposited at DDBJ/ENA/GenBank under the accession numbers JALMDJ000000000 and JALMDK000000000, for the *Crossiella* sp. strains Cross-1 and Cross-2, respectively.

## 3. Results and Discussion

### 3.1. Caves as a Promising Source of Antimicrobial Compounds (ACs)

We sampled karstic caves in the Iberian Peninsula as well as volcanic caves in the Canary Islands (Figure 1). The samples obtained from the rock surfaces and macroscopic biofilms colonizing the walls were inoculated on Petri plates with different culture media. In addition, the bacteria were collected from the air with a high-volume sampler [47]. These samplings composed a collection of more than one thousand bacteria to be tested.

We used several hundreds of isolates from different caves (Figure 1) to test for the production of ACs in a recent screen carried out as part of a European project aiming to prospect subsurface environments for bioactive compounds with potential for use in medicine, agriculture and environment. The high number of AC-producing bacteria was noticeable, from not only the Altamira and Tito Bustillo caves, but also the volcanic caves located in the Canary Islands (Table 1). Other screened marine and terrestrial caves showed <1 to 7% AC-producing strains. It is worth noting that the bacteria collected from biofilms and/or rock surfaces showed higher bioactivity than those isolated from the air, which rarely reached 10% AC-producing strains. These data suggest an interest in exploring northern Spain and volcanic caves in search of ACs.

One factor that seems to be associated with the bioactivity of the isolated bacteria should be highlighted. The characteristics of the samples from which the bacteria were originally isolated determined their capacity to develop a dynamic secondary metabolism, as was the case for the bacteria isolated from the rock surfaces compared to the airborne bacteria, independent of the number of tested bacteria (Figure S1, Supplementary Materials). Although most of the bioactive bacteria were mainly grouped into the phyla *Actinobacteria*, *Firmicutes* and *Proteobacteria*, the strains isolated from rock surfaces and air had different abilities to inhibit the growth of pathogens, suggesting that the biosynthesis of antimicrobials is a fundamental mechanism for cave wall colonization, along with other mechanisms of adaptation to those extreme environments.

### 3.2. The Case of Altamira Cave

A few authors hypothesized that cave oligotrophy enhanced the competition among the bacteria, as well as the synthesis of the ACs that inhibit the growth of other microorganisms [13,48]. This may not be the case in Altamira Cave. The cave, in addition to the massive numbers of visitors and the input of organic carbon from those visitors over 150 years, was also contaminated with drip waters containing dissolved organic matter from cattle dung from livestock just above the cave. Cuezva et al. [19] reported that although the cattle activities were discontinued in 1998, organic matter and humus from manure produced over decades entered the cave in the drip waters and sustained microorganisms. 

The most intriguing finding was the absence of fungi on the walls and sediments in the highly anthropized Altamira Cave, as determined by the failure to amplify fungal DNA from the wall samples [49], despite the high concentration of fungal spores measured in the air, which amounted up to 390 CFU/m^3^ in the Polychrome Hall [50]. This lack of fungal colonization was attributed to the *Actinobacteria* thriving on the walls, such as *Actinomadura, Amycolatopsis, Nocardia, Micromonospora, Rhodococcus* and *Streptomyces* [15,31], that produced ACs with antifungal properties. Most of these bacteria were isolated from white colonies distributed all around the cave (Figure S3, Supplementary Materials).

Bérdy [51] listed the number of species of *Actinobacteria* producing ACs, which amounted to more than 11,100 and were mainly represented by *Actinomadura* (345), *Amycolatopsis* (120), *Nocardia* (357), *Micromonospora* (740), *Rhodococcus* (13) and *Streptomyces* (~8000). Most of these genera were observed in Altamira Cave, as well as other species of the genera *Bacillus, Myxococcus, Pseudomonas* and *Stenotrophomonas*, that produce ACs with antifungal properties [15,31]. Therefore, the absence of fungi on the Altamira Cave walls was not surprising, and the conclusion was that the original cave microbiome protected the walls from secondary colonizations. 

### 3.3. Isolation of Crossiella Strains

The microbiological studies carried out between 1996 and 2012 in Altamira Cave yielded a wide collection of bacteria that were preserved in a lab collection and kept at −80 °C in a freezer [15,31,50]. The survey and screening of this collection yielded an abundance of *Streptomyces* (*S. lunaelactis, S. avidinii, S. xanthophaeus, S. xanthochromogenes, S. nojiriensis,* etc.) which predominates over other *Actinobacteria* genera (*Allokutzneria, Micromonospora, Knoellia, Rhodococcus, Crossiella, Kocuria, Kribbella, Nocardiopsis, Microbacterium,* etc.). Of all of the isolated bacteria, a total of 12 strains identified as *Crossiella* sp., with two different morphologies, stand out (Figure S4, Supplementary Materials). These strains, that inhibit the growth of pathogens, were isolated exclusively from the walls of Altamira Cave and were selected for further studies.

### 3.4. In Vitro Analyses for Inhibition of Pathogenic Microorganisms

**Figure 2:**
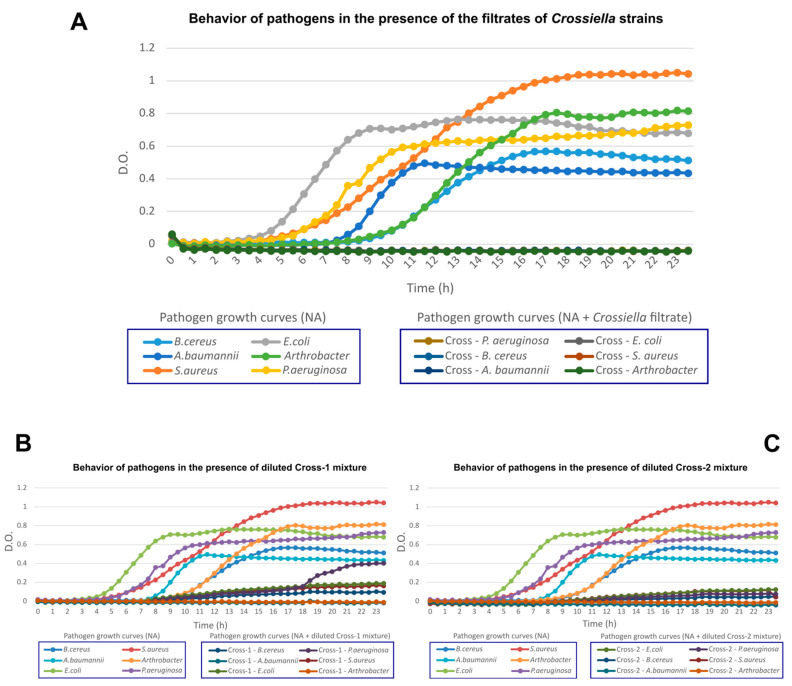
Microplate growth inhibition assay of bacterial pathogens. Pathogen inhibition tests using the filtrate of both *Crossiella* strains (**A**); diluted Cross-1 mixture (50% filtrate + 50% culture medium) (**B**); diluted Cross-2 mixture (50% filtrate + 50% culture medium) (**C**). Charts represent the growth curve of pathogens after 24 h of incubation at 30 °C (*B. cereus, S. aureus, E. coli, P. aeruginosa, A. baumannii* and *Arthrobacter* sp.) and pathogen inhibition when filtrates from *Crossiella* strains were used (Cross—*B. cereus;* Cross—*S. aureus;* Cross—*E. coli;* Cross—*P. aeruginosa;* Cross—*A. baumannii;* Cross—*Arthrobacter* sp.).

In vitro analyses were carried out with the two *Crossiella* strains to identify the distinctive features related to the inhibition of pathogenic bacteria and fungi. Both of the strains were able to inhibit the pathogens *Bacillus cereus, Staphylococcus aureus, Escherichia coli, Pseudomonas aeruginosa* and *Acinetobacter baumannii*, as well as the fungi *Aspergillus versicolor, Penicillium chrysogenum, Cladosporium cladosporioides, Fusarium solani* and *Ochroconis lascauxensis*, isolated from the caves [38,39,52]. To evaluate the ability of the *Crossiella* strains to inhibit pathogenic bacteria, an assay was performed using the filtrate with seven days of incubation. In this case, both of the strains completely inhibited the growth of the pathogens, whereas a bacteriostatic effect was observed when the filtrate was diluted to half with the culture medium (Figure 2).

**Figure 3:**
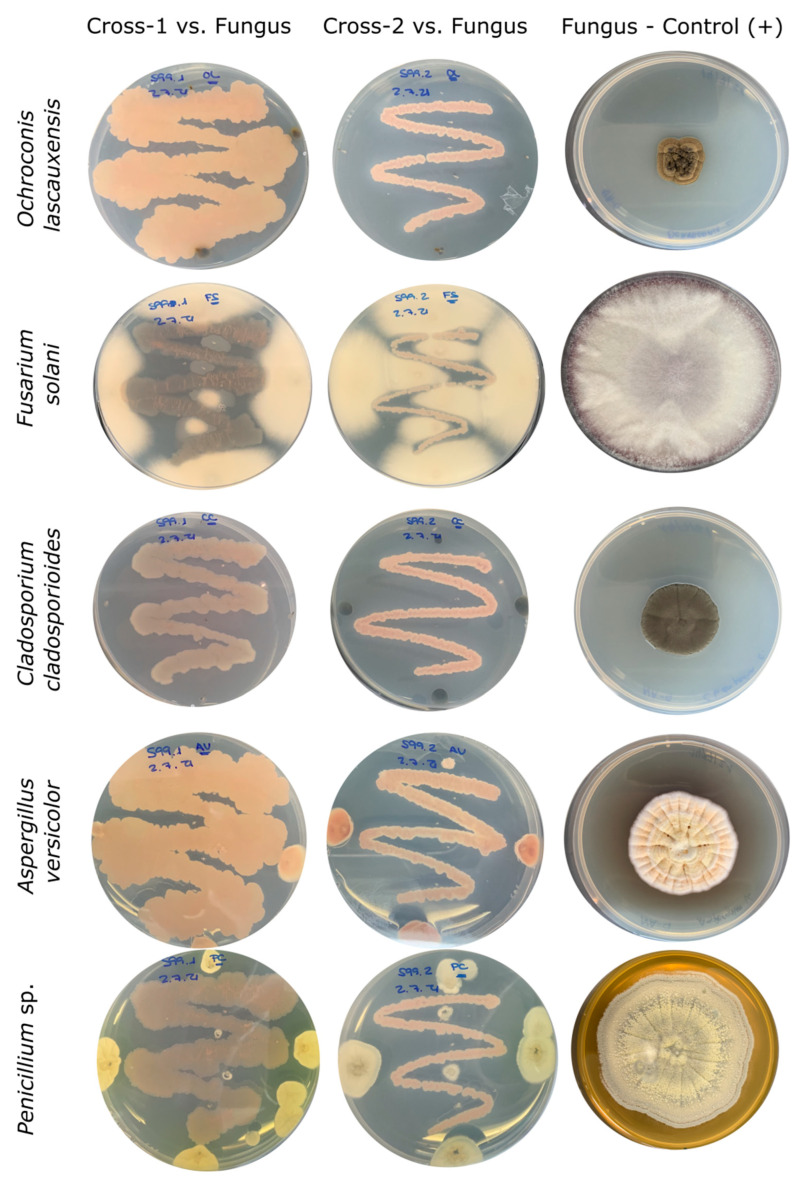
Fungal inhibition assay after 28 days of incubation. The bioactivity of strains Cross-1 (**left** column) and Cross-2 (**center** column) were evaluated in comparison with the normal growth of fungus (**right** column). Bacteria were cultured 24 h before the inoculation of fungal strains. The fungi were picked with a wood-stick and positioned separately in four external points of the plate and between the traced bacteria.

The growth inhibition in fungi was absolutely effective for all of the strains tested, except for *Fusarium solani*, for which the inhibition was minimal with respect to the rest of the fungal strains (Figure 3).

There are only two described species within the genus *Crossiella*, *C. equi* and *C. cryophila*. The first one was originally isolated from equine placentas [53], and it was associated with infectious diseases and disorders in horses [54], but no bioactive compound has been described thus far. *Crossiella cryophila*, originally described and validly published by Labeda and Lechevalier [55], within the genus *Saccharothrix*, was accommodated to the new genus *Crossiella* as the type species of this group [56]. *Crossiella cryophila* synthesizes dopsisamine, a broad spectrum antibiotic [57]. However, this antimicrobial compound does not affect *Serratia* or *Pseudomonas*.

### 3.5. In Silico Analyses for the Identification of Biosynthetic Gene Clusters

#### 3.5.1. Phylogenomics

**Figure 4:**
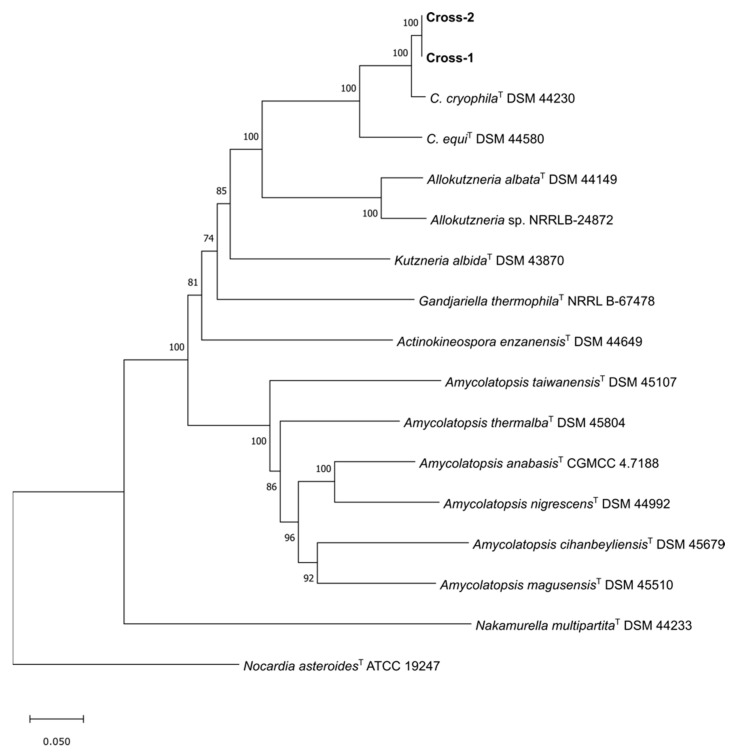
Maximum-likelihood phylogenetic tree based on 71 single-genes sequences showing the relationship of strains Cross-1 and Cross-2 with related species belonging to the family *Pseudonocardiaceae*. The bootstrap consensus tree inferred from 1000 replicates is taken to represent the evolutionary history of the taxa analyzed. There were a total of 45,445 positions in the final dataset. The genome of *Nocardia asteroides* ATCC 19247^T^ was used as outgroup. Bar, 0.05 substitutions per nucleotide position.

In silico analyses were performed to find the similarities and dissimilarities with the closest relatives of *Crossiella*. The assembly of the raw data from the sequenced genomic DNA resulted in 10,680,392 bp and 10,681,678 bp genomes and 45 and 43 contigs, for Cross-1 and Cross-2, respectively. Both of the genomes presented the same GC content of 70.4%. The phylogenetic tree showed that the closest relative was *C. cryophila*, forming, along with *C. equi,* a separated branch from the species belonging to the family *Pseudonocardiaceae* (Figure 4). A pairwise genome comparison, using the Jspecies web tool, showed values for ANIb and ANIm above 99.99%, indicating that these two strains belong to the same species of bacterium. However, values below 95% were found for the closest relatives, *C. cryophila* and *C. equi*, and suggested that the studied strains could represent a new species within the genus *Crossiella*.

#### 3.5.2. Functional Annotations

To characterize the genome through the functional enrichment of the predicted genes, two strategies were carried out to compare the presence/absence of genes in the four bacteria, Cross-1, Cross-2, *C. cryophila* and *C. equi*. The prediction and functional annotations of genes were implemented using prokka, and the antiSMASH web tool was used to predict the clusters involved in the secondary metabolism.

The gene prediction resulted in a higher number of features in Cross-2, with 9465 genes, in contrast to *C. equi*, with 8329 genes. A total of 9459 genes were predicted for Cross-1, and 8647 genes were predicted for *C. cryophila*. Likewise, Cross-2 presented a higher number of gene clusters involved in the production of secondary metabolites, with 50 predicted gene clusters, although eight of them were on the edge or in unfinished regions, due to the fragmented draft genome.

Cross-1 presented 49 clusters, seven of which were partially defined. Despite this variation between these two strains, Cross-1 and Cross-2 showed similar types and numbers of features involved in secondary metabolism. The majority of these predicted gene clusters were also found in *C. cryophila*, whereas only 13 of them were unique to the studied strains, including lasso peptides, lanthipeptides, sactipeptides, furans, polyketide synthases (PKSs) and nonribosomal peptide synthetases (NRPS). The closest sequences to the predicted gene clusters were identified in the different *Actinobacteria*, mainly species of the genera *Streptomyces, Amycolatopsis* and *Saccharotrix*, but also *Nocardiopsis, Micromonospora, Actinoplanes, Thermobifida, Actinokineospora, Rubrobacter, Blastococcus, Umezawaea, Kutzneria* and *Lentzea* (Table 2).

The lanthipeptides, or lantibiotics when referring to those compounds with antimicrobial activity, are grouped within the ribosomally synthesized and post-translationally modified peptide products (RiPPs) [58]. These compounds are divided into five classes, with lanthipeptides I and II being the most frequent, and presenting a higher antibacterial activity, and Class III and Class IV being more infrequent. The first lanthipeptide class V, lexapeptide A, was identified in 2020 [59].

Lanthipeptides are mainly active against the Gram-positive bacteria, showing a weak inhibition of the Gram-negative bacterial growth [60]. Four unknown clusters in *C. cryophila* and *C. equi*, identified as lanthipeptide classes I, III and V, were found in the Cross-1 and Cross-2 strains. The gene cluster containing lanthipeptide class I is made up of three core biosynthetic genes, showing a similarity between 91% and 94% with the sequences from the *Amycolatopsis marina* strain CGMCC 4.3568. There is no reference for antibiotics produced by this species thus far, but the genus *Amycolatopsis* is a prolific group that synthesizes bioactive compounds, especially the glycopeptide and polyketide antibiotics, within the phylum *Actinobacteria* [61]. The proposed cluster containing lanthipeptide class III, which is formed by one biosynthetic core gene, presented a similarity of 50.2% with the protein sequence of a lanthipeptide synthetase in *Streptomyces curacoi*. This species is well known because of its synthesis of curamycin, an antibiotic against Gram-positive bacteria. The predicted gene cluster for the lanthipeptide class V consisted of nine genes, of which three were identified as biosynthetic core genes. The closest sequences in the database presented very low similarity scores for two out of three biosynthetic genes located on the upstream side of the cluster, with values of 29.9% and 31.1%. These genes were originally found in *Rubrobacter xylanophilus* and *Blastococcus* sp., whereas the first three genes, including the remaining biosynthetic gene, showed a very high similarity, between 88.4% and 95.9% with the sequences found in *C. cryophila*.

Similar to the lanthipeptides, the sactipeptides are included within the RiPPs. This family is considered a promising source of new antimicrobials and other bioactive compounds, but few sactipeptides have been discovered to date, and all of them are synthesized by members of the phylum *Firmicutes* [62]. The predicted biosynthetic gene involved in this metabolite was related to a sequence from *Micromonospora ureilytica*, with a similarity of 49.2%. The family of the synthesized protein (Type II CAAX prenyl endopeptidase Rce1-like) related it to the sactipeptide described as sporulation killing factor (SkfC) in *Bacillus subtilis* [63].

The lasso peptides are a class of RiPPs as well, being grouped into four classes that are delineated based on their hydrophilicity. The lasso peptides belonging to class II are mainly produced by *Proteobacteria*, while the lasso peptides grouped into classes I, III and IV have mostly been found in *Actinobacteria*. These compounds have a wide range of activity against pathogens, but those that inhibit Gram-negative pathogens are produced by other Gram-negative bacteria [64,65,66]. For Cross-1 and Cross-2, the antiSMASH predicted four clusters involved in the synthesis of the lasso peptides (with one in an unfinished or contig edge region). Lasso peptide (1) consisted of two biosynthetic gene clusters whose closest sequences were related to *Nocardiopsis alba* and *Actinokineospora iranica*, with a similarity of 52.3% and 50.2%, respectively. These sequences were classified into the family containing ikarugamycin macrolactam cyclase, a member of the polycyclic tetramate macrolactams (PTMs), which produce a wide spectrum of natural compounds with antimicrobial activity [67,68]. Lasso peptide (2) is defined by three genes, two of which are predicted to be core biosynthetic genes. BLAST against the UniProt database resulted in similarities between 50% and 55% for the two strains of *Streptomyces*. According to antiSMASH, the lasso peptide citrulasin E was the closest metabolite. This compound was discovered after genome-mining analyses, leading to its isolation, purification and subsequent antimicrobial analysis where only the inhibition of the Gram-positive bacteria *Enterococcus faecium, Bacillus subtilis, Bacillus anthracis* and *Mycobacterium smegmatis* was observed [69]. The predicted gene cluster lasso peptide (3) was formed by two genes whose closest relative was *Saccharothrix espanaensis*, with a similarity of 63.4% and 51.1%. The fourth lasso peptide (4) cluster consisted of four genes, of which three were considered core genes. The sequences had a similarity between 51.4% and 53.4% with those from *Amycolatopsis rhizosphaerae*.

The NRPSs are responsible for the synthesis of the complex secondary metabolites in bacteria, cyanobacteria and fungi. These metabolites make up a large collection of the bioactive compounds with a wide range of activities, such as antibiotic, antifungal, antitumor and immunosuppressive agents. Two of the NRPSs were predicted to be exclusive to strains Cross-1 and Cross-2. The gene cluster NRPS (1) was formed by 17 genes, two of which were assessed as core biosynthetic genes. This cluster could be considered the union of two sets of sequences, since 11 out of the 17 predicted genes were related to the sequences found in *C. cryophila*, with similarities above 90%, and the remaining six genes were related to sequences from an *Actinoplanes* sp. strain. The gene cluster NRPS (2) consisted of one biosynthetic core gene, which was closely related to a carrier protein (CP) sequence from a *Streptomyces* sp. strain. The CP protein domains are essential components for the synthesis of nonribosomal peptides, especially siderophores [70].

The strains Cross-1 and Cross-2 also presented a set of gene clusters involved in the production of polyketide metabolites. These metabolites, which are produced by plants, fungi and bacteria, have been widely used in medicine, acting as antibiotics, antitumor, agents, antifungals, etc. [71]. They are synthesized by polyketide synthases (PKSs), which are classified as types I, II and III, depending on their structures and domains [72,73].

Up to four type I PKSs were exclusively identified in Cross-1 and Cross-2. T1PKSs (1), which is composed of one biosynthetic core gene, is related to *Streptomyces nitrosporeus*, which has been widely studied due to its antiviral and antibiotic activity against Gram-positive bacteria [74,75]. The most closely described gene cluster was pactamycin, an antitumor antibiotic synthesized by *Streptomyces pactum* [76]. It is worth noting that the genes up- and downstream of the core biosynthetic gene were closely related to *S. pactum*, with an average similarity of approximately 80%. Another polyketide synthase gene cluster, T1PKSs (2), appeared to be on a contig edge; therefore, the gene cluster could be incomplete. It was composed of four genes, two of which were identified as biosynthetic core genes that were closely related to a *Streptomyces* sp. strain. In this case, the closest known cluster was lobophorin, belonging to the group of spirotetronate natural products with strong antibacterial and antitumor behaviors [77]. T1PKSs (3) was also predicted on a contig edge and consisted of a cluster of 12 genes, three of which were considered to be core biosynthetic genes related to three species of the genera *Umezawaea, Kutzneria* and *Lentzea*, affiliated to the family *Pseudonocardiaceae*, to which *Crossiella* also belongs. The closest known cluster was lasalocid, an antibiotic against Gram-positive bacteria and antitumoral included in the class carboxyl polyether ionophores (CPIs), which were originally produced by *Streptomyces lasaliensis* [78,79,80]. The last polyketide synthase cluster, T1PKSs (4) contains the fewest genes (three) and is located on a contig edge; this cluster could be part of cluster T1PKSs (3), since the most similar known cluster was also the antibiotic, lasalocid. In this case, the closest relative to these sequences was *Streptomyces versipellis*, which synthesizes the macrocyclic compound versipelostatin, with antitumoral activity [81].

## 4. Conclusions

*Crossiella*, a rare actinobacterial genus, seems to be a source of new bioactive compounds. *Crossiella cryophila*, the closest relative of the two novel *Crossiella* strains, studied here, produces the antibiotic dopsisamine, which is not effective against *Pseudomonas aeruginosa*. The results obtained in vitro and in silico suggest that the two novel strains have different mechanisms against all of the tested pathogens, including *P. aeruginosa*. The majority of the predicted NRPS and T1PKSs gene clusters seem to suggest the synthesis of antimicrobials with activity mostly against Gram-positive bacteria, but the exclusive presence of clusters described as lasso peptides and sactipeptides indicates that these new strains of *Crossiella* are able to produce new broad-spectrum antibiotics. Another possibility that could explain the inhibition of the bacteria and fungi tested would be the synergistic effect of the different compounds synthesized by the Cross-1 and Cross-2 strains. Finally, this work shows that the subterranean ecosystems are a promising source for prospecting novel bioactive molecules. The extraction, separation and testing of each secondary metabolite should confirm whether the two *Crossiella* strains merit further research.

## Figures and Tables

**Table 1:** Cave bacteria producing antimicrobial compounds (AC) against pathogenic bacteria.

Source	Tested Strains	AC Strains	%
Altamira Cave (rock)	289	77	26.6
Tito Bustillo Cave (rock)	181	42	23.2
Ardales Cave (air)	89	9	10.1
Maravillas Grotta (air)	200	14	7.0
Tesoro Cave (air)	89	4	4.5
Tesoro Cave (rock)	8	2	25.0
La Palma Island lava tubes (rock)	160	30	23.8
Viento Cave (Tenerife Island) (rock)	31	7	22.8
Total	1047	178	17.0
Total (rocks)	669	151	22.6
Total (air)	378	27	7.1

**Table 2:** Predicted gene clusters found exclusively in *Crossiella* strains Cross-1 and Cross-2.

Cluster Type	Closest Species	Similarity (%)
Lanthipeptide-class-I	*Amycolatopsis marina*	91–92–93.8
Sactipeptide	*Micromonospora ureilytica*	49.2
Lasso peptide (1)	*Nocardiopsis alba-Actinokineospora iranica*	52.3–50.4
Furan	*Thermobifida halotolerans*	41.3
Lasso peptide (2)	*Streptomyces* sp.	55–50
Lanthipeptide-class-V	*Crossiella cryophila-Rubrobacter xylanophilus-Blastococcus* sp.	88.4–29.9–31.1
Lasso peptide (3)	*Saccharothrix espanaensis*	63.4–51.1
NRPS (1)	*Actinoplanes* sp.-*Crossiella cryophila*	56.3–91.2
T1PKSs (1)	*Streptomyces nitrosporeus*	53.9
Lanthipeptide-class-III	*Streptomyces curacoi*	50.2
NRPS (2)	*Streptomyces* sp.	53.1
T1PKSs (2) (Region on contig edge)	*Streptomyces* sp.	49.1–48.9
T1PKSs (3) (Region on contig edge)	*Umezawaea tangerina*-*Kutzneria buriramensis*-*Lentzea waywayandensis*	65–49.7–52.7
Lasso peptide (4) (Region on contig edge)	*Amycolatopsis rhizosphaerae*	52.4–53.4–51.4
T1PKSs (4) (Region on contig edge)	*Streptomyces versipellis*	55.3–50

Prediction and identification of clusters using antiSMASH (“Cluster type”) and UniProt BLAST with UniProtKB database (“Closest species” and “Similarity”). Results showed in “Closest species” and “Similarity” belong to the core biosynthetic genes predicted by antiSMASH. NRPS: Non-Ribosomal Peptide Synthetase; T1PKSs: Polyketide Synthase Type 1.

## Data Availability

The sequencing data generated in this study were deposited to the National Center for Biotechnology Information (NCBI) under the BioProject ID number PRJNA769239. The whole-genome shotgun project has been deposited at DDBJ/ENA/GenBank under the accession number JALMDJ000000000 and JALMDK000000000, for *Crossiella* sp. strains Cross-1 and Cross-2, respectively. The GenBank/EMBL/DDBJ accession numbers of 16S rRNA gene sequences of strains Cross-1 and Cross-2 were ON669108 and ON669109, respectively.

## Supplementary Materials

The following supporting information can be downloaded at: https://www.mdpi.com/article/10.3390/microorganisms10081575/s1, Figure S1: Number of strains tested in each cave and strains producing bioactive compounds; Figure S2: Number of cave isolates that produced bioactive compounds inhibiting each target bacterium; Figure S3: Spatial distribution of white colonies on Altamira Cave ceiling and walls. The Polychrome Hall features bison, a deer, and other animals, painted more than 14,000 years ago and spread across 150 m^2^ of ceiling; Figure S4: Cultures of two strains of *Crossiella* sp. isolated from white colonies dwelling in the walls of Altamira Cave. Both strains were cultured in nutrient agar and incubated for seven days at 28 °C.
