# Supplementary material for: The Rare Actinobacterium Crossiella sp. Is a Potential Source of New Bioactive Compounds with Activity against Bacteria and Fungi
Source: Microorganisms. 2022 Aug 5;10(8):1575. doi: 10.3390/microorganisms10081575 (PMC9415966; doi:10.3390/microorganisms10081575)
Supplement: Supplementary file 1 [file microorganisms-10-01575-s001.zip › microorganisms-1826917-supplementary.pdf]

# SUPPLEMENTARY MATERIALS

## The Rare Actinobacterium *Crossiella* sp. is a Potential Source of New Bioactive Compounds with Activity against Bacteria and Fungi

**Jose Luis Gonzalez-Pimentel, Irene Dominguez-Moñino, Valme Jurado, Leonila Laiz, Ana Teresa Caldeira, and Cesareo Saiz-Jimenez**

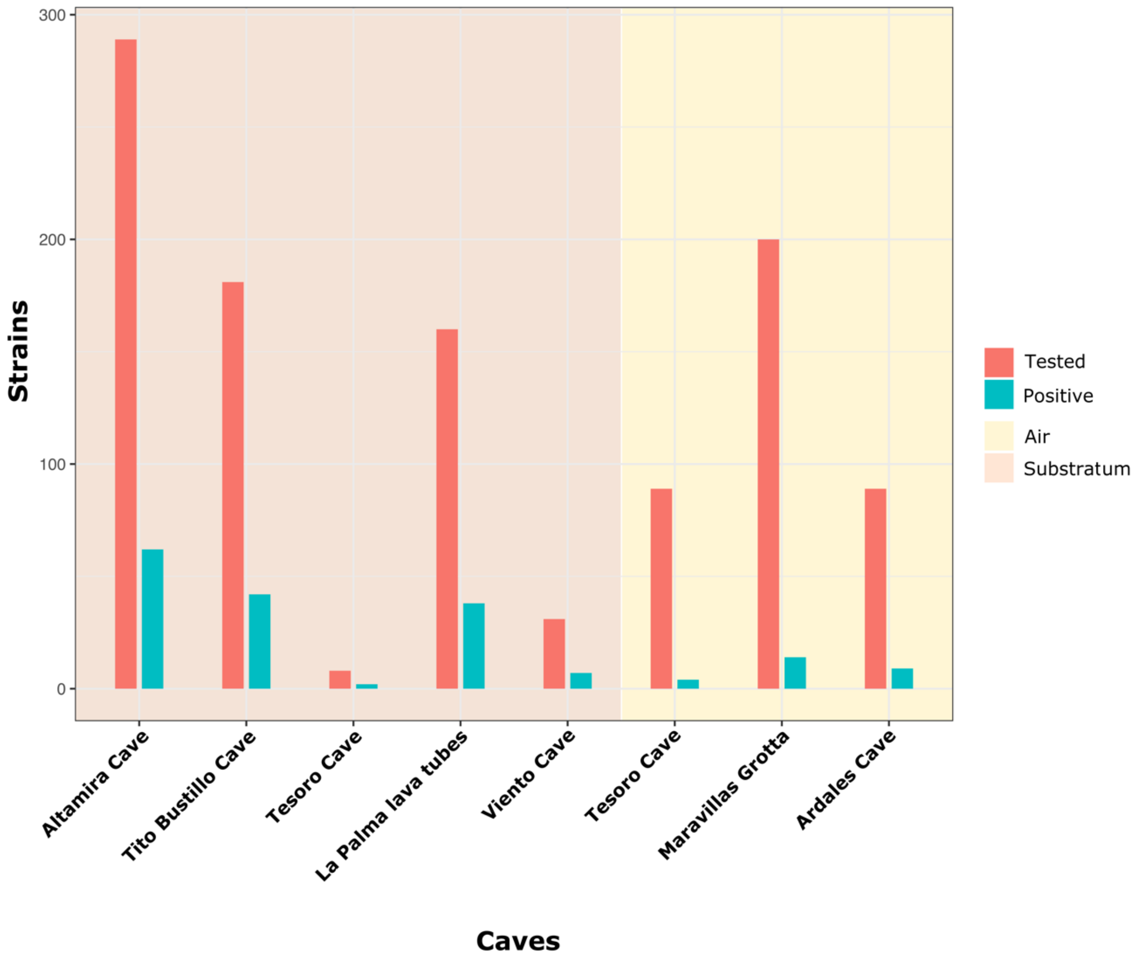


**Figure S1**. Number of strains tested in each cave and strains producing bioactive compounds.

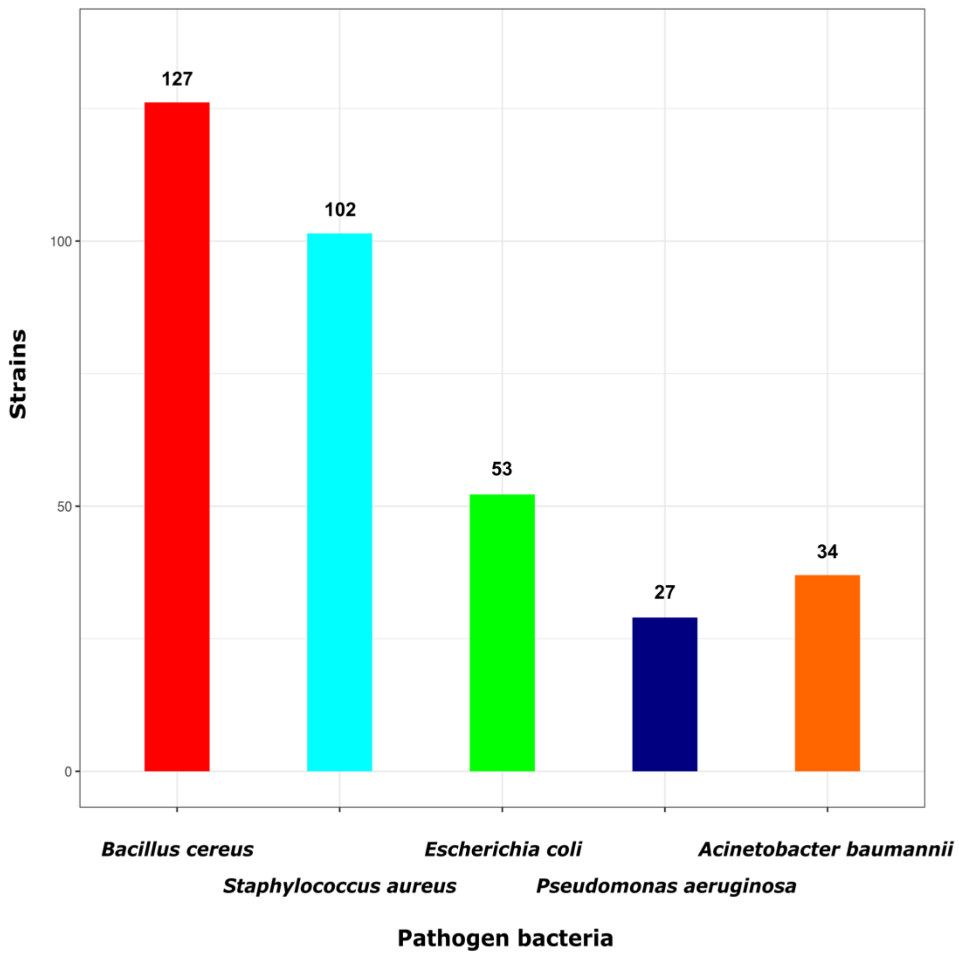


**Figure S2**. Number of cave isolates that produced bioactive compounds inhibiting each target bacterium.

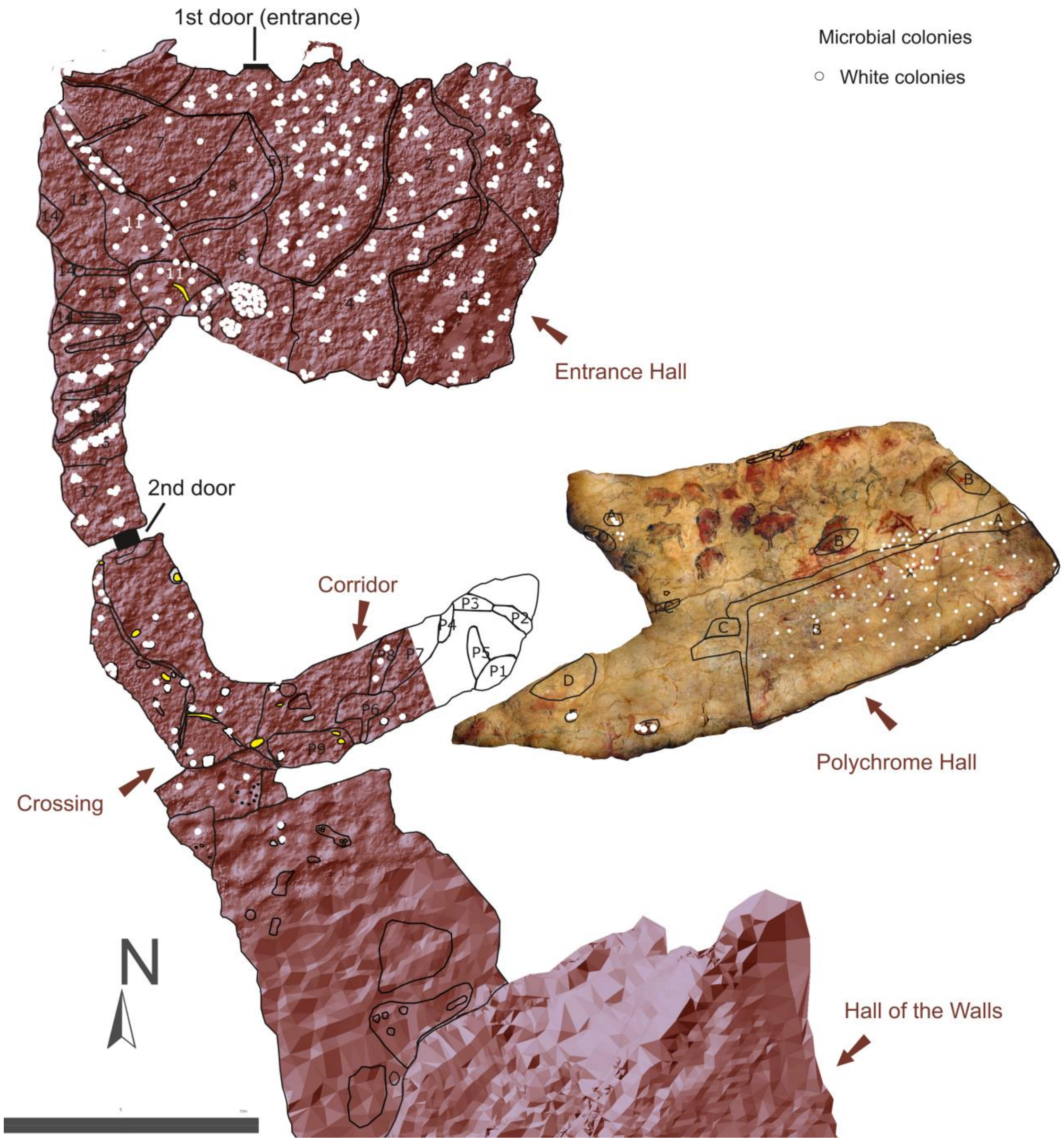


**Figure S3.** Spatial distribution of white colonies on Altamira Cave ceiling and walls. The Polychrome Hall features bisons, a deer, and other animals, painted more than 14,000 years ago and spread across 150 m² of ceiling.

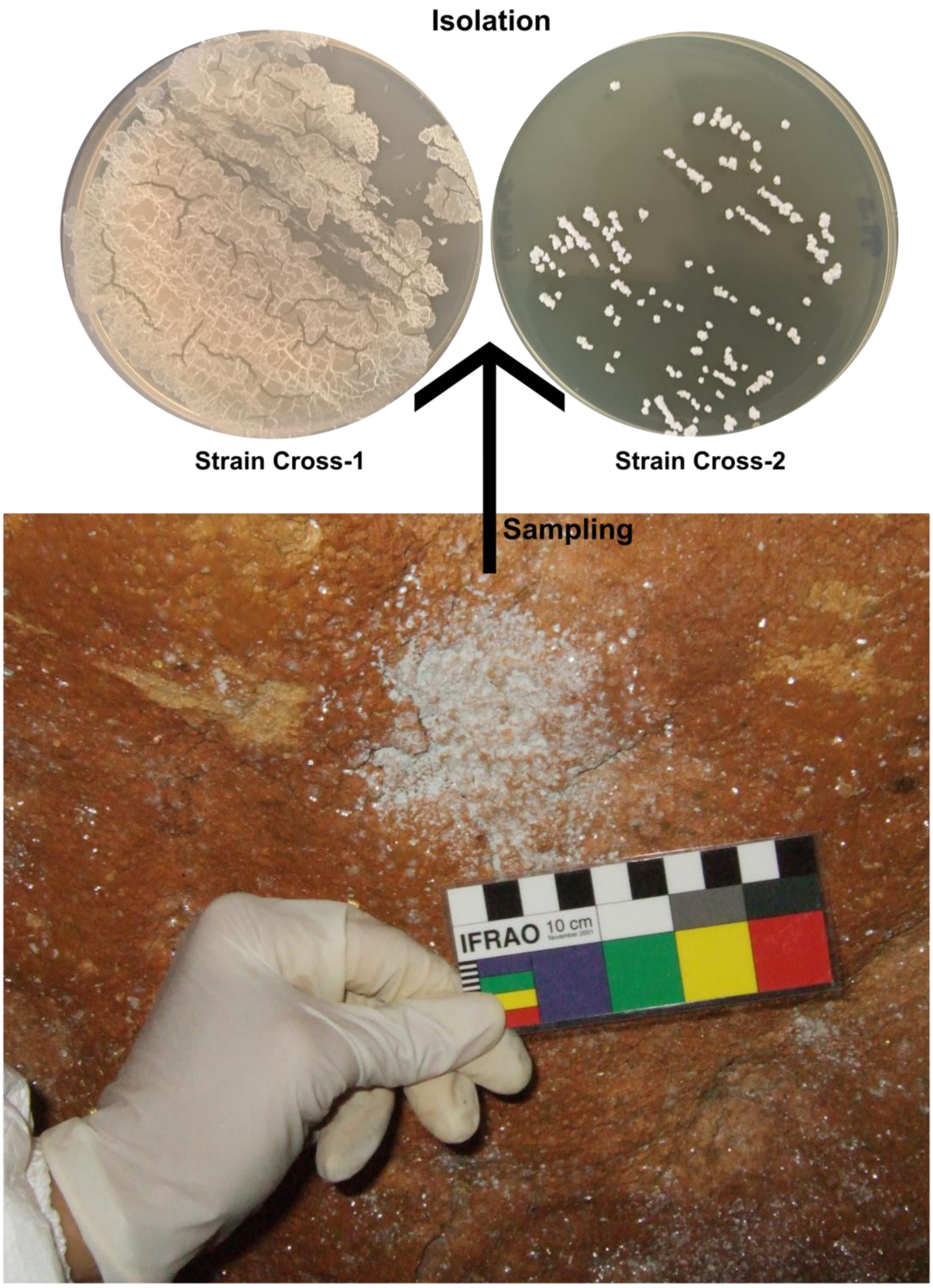


**Figure S4.** Cultures of two strains of *Crossiella* sp. isolated from white colonies dwelling in the walls of Altamira Cave. Both strains were cultured in nutrient agar and incubated for seven days at 28°C.
